# Effects of a rework program in a university hospital and predictors of work restoration and maintenance in the participants

**DOI:** 10.3389/fpsyt.2022.944472

**Published:** 2022-07-25

**Authors:** Hitomi Yamashita, Akari Sakai, Takeshi Terao

**Affiliations:** ^1^Department of Neuropsychiatry, Oita University Faculty of Medicine, Yufu, Japan; ^2^Tsurumidai Hospital, Beppu, Japan

**Keywords:** psychiatric rehabilitation, rework program, work restoration, work maintenance, trail making test, depression, bipolar disorder

## Abstract

During sickness absence, it appears necessary for psychiatric patients suffering from depression or bipolar disorder to undergo a psychiatric rehabilitation called the rework program that aids in work restoration and maintenance. However, few studies have investigated the effects of such a program and predictors of work restoration and maintenance in the participants. Here, we aimed to investigate the effects of a rework program as well as to examine whether cognitive function and mental state at the end of the rework program predict the probability of work restoration and maintenance and whether the frequency of rework program participation predicts successful work restoration and maintenance. The rework program included both patients absent from work and unemployed patients. Patients completed assessments including Trail Making Test Type B (TMT-B), the Wisconsin Card Sorting Test, and the Social Adaptation Self-Evaluation Scale just before graduating from the rework program. Simultaneously, their depressive state was assessed using the Hamilton Depression Rating scale. The patients were divided into the job group, comprising 94 patients who were able to restore their work or get a new job, and the non-job group, comprising 34 patients who were not able to do so. We found that the program was more effective in patients absent from work than in unemployed patients, TMT-B could predict work restoration and maintenance, and the frequency of rework program participation could predict work restoration but not work maintenance. Based on the findings, we propose “Yamashita’s criterion” where a TMT-B completion time of 70 s is the cut-off point for work restoration. The present findings may provide useful evidence that could aid in the further development of rework program(s).

## Introduction

Employees who are on sick leave due to common mental health disorders, such as depression, face difficulties in restoring their work. Recurrent sickness absence episodes may often be more serious and long lasting than the first sickness absence episode. In addition, frequent sickness absence is related to an increased risk of work disability (1). A variety of interventions have been implemented for reducing sickness absence. A recent meta-analysis (2) showed that a combination of a work-directed intervention and clinical intervention reduced the number of sickness absence days; however, this effect was not seen beyond a 1-year period. In addition, it showed that this combination improved depressive symptoms and probably increased work functioning to a greater extent than the administration of routine care. Further, the review (2) revealed that the effectiveness of specific work-directed interventions was not greater than that of the sole administration of routine work-directed care, whereas psychological interventions reduced the number of sickness absence days more effectively than routine care. Finally, it showed that compared with routine care, interventions for the improvement of clinical care mitigated sickness absence and depression, and that there was no evidence suggesting that the effects of one antidepressant medication on sickness absence were different from those of another (2). These findings suggest that comprehensive interventions rather than specific work-directed interventions may be useful for reducing sickness absence.

Very recently, another meta-analysis (3) was performed to identify factors predicting work restoration in individuals on sick leave due to stress, anxiety, and depression. The review showed that predictors decreasing the probability of work restoration at any time point were higher age, being male, neuroticism and openness, previous sickness absence, and higher symptom scores, whereas predictors increasing the probability of work restoration were positive work restoration expectations, high work restoration self-efficacy, general self-efficacy, conscientiousness, and a high workability index (3). In addition, the review revealed that work restoration within 3 months of sick leave was associated with positive work restoration expectations, while the probability of work restoration after 12 months of sick leave increased with higher education levels (3). It should be noted that these predictors were mainly identified in observational cohort studies that did not involve interventions for work restoration.

Specifically, it is important that help is provided to facilitate work restoration after the recovery from depression. For this purpose, we recently managed a Research Topic collection entitled “How to Help Employees Returning to Work Following Depression” (4). In this Research Topic collection, first, Dewa et al. (5) emphasized the importance of workplace educational programs and interventions for addressing workplace stigma. Such programs and interventions are necessary and may encourage workers to disclose their mental health problems to managers with the certainty that they would receive warm-hearted support in their workplaces. Second, in Japan, there is a comprehensive program called the “Rework Program” which involves “an intervention program for work restoration.” As part of this rework program, employees on sick leave visit the hospital or clinic in the morning, which simulates the commute to office. During the early stage, participants enjoy light sports and recreation. During the advanced stage, they participate in simulated office work, light work, psychoeducation, and psychological therapies such as cognitive behavioral therapy (CBT). Moreover, during the course of the program, they write a report that involves them looking back over the situation surrounding their absence from work and preparing for the next work restoration. Regarding the usefulness of this rework program, Ohki et al. (6) showed that the duration of work maintenance after restoration was significantly longer in subjects undergoing the rework program along with routine treatment than in subjects solely receiving routine treatment, suggesting that the rework program is effective in improving work maintenance. Regarding the predictors of work restoration after a rework program, Hoaki and Terao (7) revealed that the frequency of participation in a rework program was positively associated with work restoration but not with work maintenance, indicating that the frequency can predict successful work restoration but not work maintenance. Third, Hayasaka et al. (8) explored factors related to work restoration within 1 year after discharge in patients with mood disorder who participated in a rework program called the occupational support program. They showed that the word count in the transcription task of the rework program and mood status after the completion of the rework program were significantly associated with the time to achieve employment restoration. Finally, a case report by Wisenthal (9) described a rework program called cognitive work hardening, which is a multi-element, work-oriented intervention for work restoration following a depressive episode. These interventions suggest the usefulness of a rework program; however, currently, scientific evidence is definitely insufficient.

In the present study, we aimed to investigate the effects of a rework program and to examine whether cognitive function and mental state at the end of the rework program predict the probability of work restoration and maintenance; further, we aimed to investigate whether the frequency of rework program participation predicts successful work restoration but not work maintenance similar to the findings of Hoaki and Terao (7).

## Materials and methods

### Rework program settings and steps

The rework program at our institution, the Oita University Hospital, was started on 15 May 2017. This program was administered from 9 a.m. to 4 p.m. with a 1-h lunch break every Monday to Friday. The average number of participants was approximately 15 per day. These participants mostly suffered from depression, bipolar disorder, or adjustment disorder; however, a small proportion suffered from schizophrenia and schizoaffective disorder. Notably, although this is a rework program, we permit unemployed patients to attend in order to aid them in gaining new employment. Usually, the rate of employed vs. unemployed participants is approximately 8:2. The program is mainly administered by an occupational therapist, a nurse, and a clinical psychologist with support from a psychiatric social worker, an art therapist, and psychiatrists.

As a step in the workflow of the rework program, immediately after the introduction, we attempt to correct distorted sleep-wake rhythms, if any, by filling in a sheet for recording daytime activities and sleep state every day. Further, the program consists of two stages: an early stage focusing on physical recovery and advanced stage focusing on work restoration. The program comprises a variety of therapeutic activities ranging from art therapy involving activities such as pottery and painting and exercise therapy involving activities such as playing table tennis to psychotherapy such as mindfulness-based interventions and CBT. In addition, patients are encouraged to participate in cognitive training, to perform simulated office work, to write self-reports by recalling the past situation leading to sick leave, and to think of possible remedial measures for preventing further sick leave. They are also instructed to participate in group discussions regarding various themes, which may aid in eliminating obstacles in their thoughts about work restoration. The duration and frequency of participation in the program depend on individual participants. In principle, a staff meeting is conducted to decide the timing of graduation from the program, during which staff members comprehensively assess a participant’s mental state, scores in the rework program, and daily lives in order to determine whether the participant can restore and maintain work.

### Subjects

In this study, the inclusion criteria were (1) psychiatric patients who entered and either completed or terminated the rework program at Oita University Hospital from 15 May 2017 to 28 December 2020, for work restoration or preparation for staring work, (2) patients who were followed-up from the start of the program until 30 June 2021, (3) patients with psychiatric diagnoses related to depression, bipolar disorder, or adjustment disorder, and (4) patients who provided informed consent to participate in this study. The exclusion criteria were (1) patients who entered the rework program for reasons apart from work restoration or preparation for staring work (e.g., those only participating in CBT for psychological therapy but not for work restoration), and (2) those with psychiatric diagnoses related to schizophrenia and schizoaffective disorder. The protocol of this study was approved by the university’s ethics committee.

Of 184 patients who entered the program from 15 May 2017 to 28 December 2020, 26 patients did not provide consent to participate in this study, 18 patients did not complete the program until 28 December 2020, and 12 patients were excluded based on the exclusion criteria. Consequently, 128 patients met the inclusion criteria and provided informed consent. As shown in Table 1, participant mean [standard deviation (SD)] age was 40.9 (10.2) years, and there were 91 men and 37 women. According to the International Statistical Classification of Diseases and Related Health Problems (ICD), the diagnoses were as follows: mood disorder (ICD = F3) (*N* = 106: 78 with depression and 28 with bipolar disorder), adjustment disorder (ICD = F43) (*N* = 18), and anxiety disorder (ICD = F4) (*N* = 4: 2 with social anxiety disorder, 1 with generalized anxiety disorder, and 1 with post-traumatic stress disorder). The employment status was as follows: unemployed (*N* = 23) and absent from work (*N* = 105). Of the 105 patients who were absent from work, their jobs were 31 bank staffs, 30 public servants, 10 clerks, 8 factory workers, 8 managers, 4 engineers, 3 teachers, 2 nurses, and 9 others. It should be noted that not only patients who were absent from work but also those who were unemployed participated in the program in this study. As for the marital status, 59 patients were married, 65 were unmarried, and the status was unknown in four patients.

**TABLE 1:** Demographic data of the subjects.

		Mean	*SD*	*N*
Age (years)		40.9	10.2	
Gender				
	Female			37
	Mele			91
Diagnoses				
	Depression			78
	Bipolar disorder			28
	Adjustment disorder			18
	Social anxiety disorder			2
	Generalized anxiety disorder			1
	Post-traumatic stress disorder			1
Employment status				
	Absent from work			105
	Unemployed			23
Jobs				
	Bank staffs			31
	Public servants			30
	Clerks			10
	Factory workers			8
	Managers			8
	Engineers			4
	Teachers			3
	Nurses			2
	Others			9
Marital status				
	Married			59
	Unmarried			65
	Unknown			4

### Assessments of cognitive function and mental state

Patients completed assessments including Trail Making Test Type B (TMT-B), the Wisconsin Card Sorting Test (WCST), and the Social Adaptation Self-Evaluation Scale (SASS) just before graduating from the rework program. Simultaneously, their depressive state was assessed using the Hamilton Depression Rating scale (HAM-D), in which scores of equal to or less than 7 are within the normal range.

The TMT provides information regarding visual search and scanning, speed of processing, mental flexibility, and executive functions. It consists of two parts: TMT-A that requires an individual to draw lines sequentially connecting 25 encircled numbers distributed on a sheet of paper and TMT-B that has similar task requirements except that the individual must alternate between numbers and letters (e.g., 1, A, 2, B, 3, C, etc.) (10). The score on each part represents the amount of time (second [s]) required to complete the task. In this study, we used the time taken to complete TMT-B, which included 13 numbers and 12 Japanese letters and was performed on a personal computer. The reason why we selected TMT-B was that TMT-B may be more difficult than TMT-A and it seems likely that TMT-B may have less ceiling effects and more appropriately estimate cognitive function than TMT-A. The higher the TMT score, the slower the speed of various processing functions.

The WCST requires participants to sort out stimulus cards according to key cards based on categories that change periodically and is mainly used to assess cognitive flexibility. Further, the WCST is a complex task that requires the use of multiple executive functions including attention, memory, and implicit learning (11). In this study, we used the number of categories achieved on a personal computer. The larger the number, the higher the level of cognitive flexibility.

The SASS is a 21-item self-evaluated scale for the evaluation of patient social motivation and behavior in the case of depression (12); this scale was translated into Japanese, and its validity and reliability were confirmed (13, 14). The higher the SASS score, the better the social function.

### Statistical analyses

As mentioned above, the program included not only patients who were absent from work but also unemployed patients. Therefore, we defined the “job group” as patients who were able to restore their job or get a new job and the “non-job group” as patients who could not restore their job or get a new job.

The depression scores assessed by HAM-D; cognitive function measured by TMT-B and WCST; social adaptation measured by SASS; and frequency of rework program participation were compared between the job and non-job groups using *t*-tests. Categorical parameters, such as gender, employment status, and marital status, were compared using χ^2^-tests (crude model). Subsequently, a binary logistic regression analysis involving the likelihood ratio method (forward selection) was performed using the job or non-job group as a dependent factor and age, gender, employment status, marital status, HAM-D scores, TMT-B scores, WCST scores, SASS scores, and frequency of rework program participation as independent factors (adjusted model).

Moreover, in the job group, the time course of work maintenance was analyzed using a Kaplan-Meier survival analysis (crude model) and Cox proportional hazards models, in which whether or not the job status was maintained (e.g., absent from work, quit the job, or lost to follow-up) was the dependent factor, and age, gender, absence from work or unemployment, TMT-B scores, WCST scores, SASS scores, and frequency of rework program participation were independent factors (adjusted model).

To perform a subclass analysis focused on patients absent from work, the unemployed patients were excluded, and the above analyses were repeated.

Finally, receiver operating characteristic (ROC) curves were drawn for potentially robust predictors in order to determine cut-off points for work restoration and maintenance.

SPSS Statistics 27 (IBM) was used for these analyses.

## Results

### Cognitive function, mental state, and frequency of rework program participation

The mean (*SD*) cognitive function scores measured by TMT-B [the amount of time (s)], the WCST (number of categories achieved), and the SASS (total scores) were 60.6 (24.2), 9.8 (1.5), and 36.2 (8.3), respectively.

The following scores were shown as references. The mean (*SD*) TMT-B score of community-dwelling individuals aged 35–44 years similar to the present subjects was 58.5 (16.4) (10). The mean (*SD*) WCST score (number of categories achieved) of neurologically healthy adults aged 19–53 years was 6.2 (1.9) (15). Finally, the mean (*SD*) SASS score of healthy Japanese individuals was 36.5 (5.7) and that of depressive patients was 32.2 (8.5). Therefore, all three mean scores of the participants in the present study were almost comparable to those of healthy individuals.

As for mental state, the mean (*SD*) HAM-D score was 3.9 (3.7), which was within the normal range (≤ 7).

In addition, the mean (*SD*) frequency of rework program participation was 64.2 (64.9). As a reference, we used the mean (*SD*) frequency of participation in another rework program, which was 67.8 (41.2) (7); this was comparable to that in the case of the participants in the present study.

### Work restoration or getting a new job

Of the 128 patients, 94 patients were included in the job group, among whom 89 patients could restore their job, and five patients could get a new job. On the other hand, 34 patients were included in the non-job group, among whom 16 patients could not restore their job, and 18 patients could not get a new job. Therefore, the proportion of patients in the job group was 94/128 × 100 = 73.4%. In the narrow sense, the proportion of patients who were absent from work and could restore their job was 89/105 × 100 = 84.8%, whereas the proportion of those who could get a new job among the unemployed patients was 5/23 × 100 = 21.7%. This difference was significant (χ^2^ = 38.4, *p* < 0.0001), and patients who were absent from work were significantly more successful in restoring their job status than unemployed patients. As a reference, we considered a previously reported restoration rate of 84% among patients absent from work (7), which was comparable to that in the present study.

As shown in Table 2, there were significant differences between the job and non-job groups in employment and marital status but not in psychiatric diagnoses or gender (significant tendency). Further, the job group had significantly lower HAM-D scores, significantly higher SASS scores, significantly shorter TMT-B completion times, and significantly larger numbers of categories achieved in the WCST. Moreover, the job group had a significantly higher frequency of rework program participation.

**TABLE 2:** Comparison of job group and non-job group.

		Job group	None-job group	*P*
Age (years)		41.0 (10.3)	40.4 (9.9)	0.77
Gender				0.066
	Female	23	14	
	Mele	71	20	
Diagnoses				0.57
	Depression	55	23	
	Bipolar disorder	20	8	
	Adjustment disorder	16	2	
	Social anxiety disorder	1	1	
	Generalized anxiety disorder	1	0	
	Post-traumatic stress disorder	1	0	
Employment status				0.001
	Absent from work	89	16	
	Unemployed	5	18	
Marital status				0.049
	Married	49	10	
	Unmarried	44	21	
HAM-D		2.9 (2.6)	6.9 (4.9)	0.001
SASS		38.3 (7.3)	30.0 (8.0)	0.001
TMT-B (seconds)		54.6 (17.7)	79.0 (31.3)	0.001
WCST		10.1 (1.0)	9.0 (2.2)	0.018
Frequency of rework (total number)		82.3 (66.5)	14.3 (16.4)	0.001

Mean (SD), HAM-D, Hamilton depression rating scale; SASS, Social Adaptation Self-Evaluation Scale; TMT-B, Trail Making Test Type B; WCST, Wisconsin Card Sorting Test. Statistically, t-test was performed for continuous variables and χ^2^-test for categorical variables.

The binary logistic regression analysis involving the likelihood ratio method (forward selection) confirmed the significance of the model and showed that employment status, SASS scores, TMT-B scores, and the frequency of rework program participation were significantly associated with the job group but not the other factors (Table 3). This implied that absence from work rather than unemployment (employment status), higher SASS scores, shorter TMT-B completion times, and higher frequency of rework program participation could predict the probability of work restoration or of getting a new job.

**TABLE 3:** The binary logistic regression analysis with likelihood ratio method (forward selection) for job group in work restoration.

Variables	Hazard ratio	95% confidence interval	*P*
Employment status	27.3	3.5–212.8	0.002
SASS	0.86	0.77–0.97	0.011
TMT-B	1.04	1.01–1.08	0.022
Frequency of rework	0.95	0.92–0.98	0.002

SASS, Social Adaptation Self-Evaluation Scale; TMT-B, Trail Making Test Type B Absence from work than unemployed state (employment status), more SASS scores, shorter TMT-B and more frequency of the rework participation could predict work restoration or getting a job.

### Work maintenance

**FIGURE 1:**
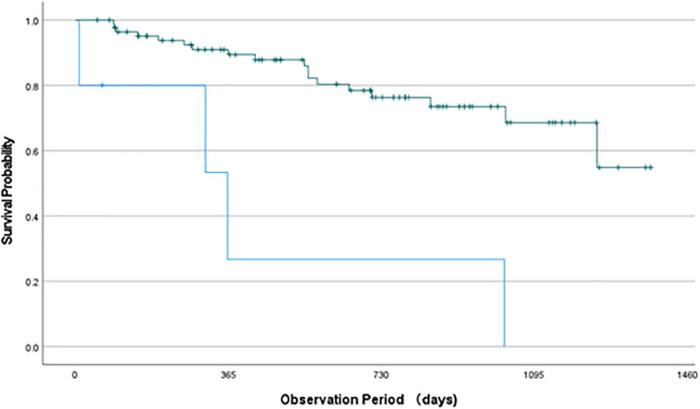
Survival curve of Kaplan-Meier survival analysis. Green line indicates the work maintenance state of patients absent from work and blue line shows that of unemployed patients. These curves were significantly different (Log-rank test; χ^2^ = 17.1, *p* < 0.001). For example, 2 years after restoration about 80% patients maintained their work (green line) whereas less than 30% patients maintained their work (blue line).

In the job group (94 patients), four patients were lost to follow-up immediately after they restored their job or got a new job. Therefore, 90 patients were followed-up from the viewpoint of work maintenance. Survival curves of patients who were absent from work and of those who were unemployed were obtained through the Kaplan-Meier survival analysis of the job group (Figure 1). The mean (SD) estimated survival time of these patients was 1,095 (55) and 454 (208) days, respectively. These curves were significantly different (log-rank test; χ^2^ = 17.1, *p* < 0.001). For example, 2 years after work restoration, approximately 80% of patients who were absent from work were able to maintain their work compared with less than 30% of the unemployed patients.

The Cox proportional hazards model with the likelihood ratio method (forward selection) confirmed the significance of the model and revealed that maintenance following work restoration was significantly associated with only the employment status (Table 4). This implies that absence from work rather than unemployment could predict work maintenance.

**TABLE 4:** The Cox proportional hazards model with likelihood ratio method (forward selection) for job group in work maintenance.

Variables	Hazard ratio	95% confidence interval	*P*
Employment status	7.28	2.4–22.0	0.001

Absence from work than unemployed state could predict work maintenance.

### Subclass analysis

To focus on patients who were absent from work, the unemployed patients were excluded, and the above analyses were repeated.

Regarding work restoration, the binary logistic regression analysis with the likelihood ratio method (forward selection) confirmed the significance of the model and revealed that HAM-D scores, TMT-B scores, and the frequency of rework program participation were significantly associated with the job group but not the other factors (Table 5). This implies that lower HAM-D scores, shorter TMT-B completion times, and higher frequency of rework program participation could predict work restoration.

**TABLE 5:** The binary logistic regression analysis with likelihood ratio method (forward selection) for restoration group in work restoration.

Variables	Hazard ratio	95% confidence interval	*P*
HAM-D	1.31	1.03–1.67	0.029
TMT-B	1.04	1.00–1.07	0.044
Frequency of rework	0.95	0.92–0.99	0.007

HAM-D, Hamilton depression rating scale; TMT-B, Trail Making Test Type B Less HAM-D scores; shorter TMT-B and more frequency of the rework participation could predict work restoration.

With regard to work maintenance, the Cox proportional hazards model with the likelihood ratio method (forward selection) confirmed the significance of the model and showed that maintenance of work restoration was significantly associated with age and TMT-B scores (Table 6). This implies that older age and shorter TMT-B completion times could predict work maintenance.

**TABLE 6:** The Cox proportional hazards model with likelihood ratio method (forward selection) for restoration group in work maintenance.

Variables	Hazard ratio	95% confidence interval	*p*
Age	0.93	0.88–0.98	0.008
TMT-B	1.03	1.00–1.05	0.034

TMT-B, Trail Making Test Type B Older age and shorter TMT-B could predict work maintenance.

### Receiver operating characteristic curves

**FIGURE 2:**
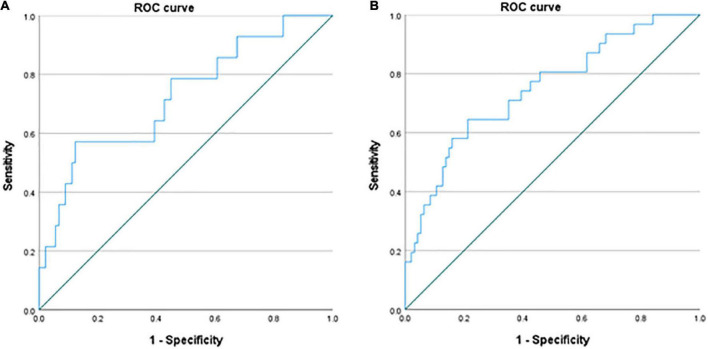
Receiver Operating Characteristic (ROC) curves of the whole samples and the restoration group. There are two ROC curves of the whole sample for work restoration using a TMT-B completion time [**(A)** upper figure; area under the curve (AUC) = 0.75, *p* < 0.001, cut-off point = 66.8 s] and the restoration group for work restoration [**(B)** lower figure; AUC = 0.73, *p* = 0.007, cut-off point = 76.5 s].

Considering the above findings, the TMT-B score appears to be a robust predictor for work restoration and maintenance. Therefore, ROC curves were drawn for TMT-B scores in order to determine a cut-off point. As shown in Figure 2, there were two ROC curves: one for work restoration in the whole sample [upper figure; area under the curve (AUC) = 0.75, *p* < 0.001, cut-off point = 66.8 s] and another for work restoration in the restoration group (lower figure; AUC = 0.73, *p* = 0.007, cut-off point = 76.5 s). However, the ROC curves for work maintenance in the whole sample or in the restoration group were not significant. Based on the findings, we proposed Yamashita’s criterion where a TMT-B completion time of 70 s is the cut-off point for work restoration.

Of the 128 patients, 125 patients had the TMT-B scores. According to Yamashita’s criterion, 77 of 90 (85.6%) patients who completed TMT-B within 70 s could restore their job or got a new job whereas 17 of 35 (48.6%) patients who completed it beyond 70 s could (χ^2^ = 18.5, *p* < 0.001). The sensitivity was 77/94 × 100 = 81.9% and the specificity was 18/33 × 100 = 58.1%.

## Discussion

The main findings of the present study were as follows: (1) unemployed patients faced more difficulties in getting a job and maintaining work than patients absent from work; (2) TMT-B scores could predict the probability of work restoration or of getting a new job in the whole sample, and TMT-B scores could predict the probability of work restoration and maintenance in the patients absent from work; and (3) the frequency of rework program participation could predict the probability of work restoration or of getting a new job but not of work maintenance in both the whole sample and patients absent from work.

Most rework program facilities accept only patients who are absent from work but not unemployed patients probably because the difficulties faced by unemployed patients are twofold: difficulties in making progress in developing their work skills from a null state *via* a rework program and difficulties in finding and getting a new job. Therefore, the significant difference between the proportion of patients absent from work who were able to restore their job (84.8%) and proportion of unemployed patients who were able to get a new job (21.7%) was within the expected range. Unexpectedly, however, the mean estimated survival time was 1,095 days in patients who were absent from work and 454 days in unemployed patients. We expected to find no significant difference in work maintenance between patients absent from work and unemployed patients once the latter started working. Although the reason is unknown, unemployed patients may be vulnerable to work and/or the workplace. If so, a special program may be required for helping unemployed patients to get and maintain a job. For example, a staff member could provide them with support even after they graduate from a rework program.

TMT-B appears to be a robust tool for the prediction of work restoration and maintenance in patients absent from work. Since the TMT can provide varied information on visual search and scanning, speed of processing, mental flexibility, and executive functions (10), the score may be associated with functions required for work restoration and maintenance. Other tests, such as the SASS and HAM-D, could predict work restoration in the whole sample and in patients absent from work, respectively. At least in the patients absent from work, the depressive state should be treated as much as possible to facilitate work restoration. Although the WCST is a complex task that requires the use of multiple executive functions, including attention, memory, and implicit learning (11), there was no association between the number of categories achieved and work restoration and maintenance. These findings suggest that not all tests could predict work restoration or maintenance, but specific ones, such as TMT-B, can predict these outcomes.

In accordance with the findings of Hoaki and Terao (7), the frequency of rework program participation could predict the probability of work restoration or of getting a new job but not of work maintenance in both the whole sample and patients absent from work. This is probably because the effects of the rework program may fade day by day. Instead of the fading effects of the rework program, as we pointed out (1), it seems important for individual workers to recognize their graduation from the rework program as a starting point and to continue to reflect on themselves in order to foster the ability of their minds to cooperate with other workers with a feeling that their job is worthwhile.

Although a meta-analysis (3) showed that the predictors that decreased the probability of work restoration at any time point were higher age and being male, our findings demonstrated that neither age nor gender could predict the probability of work restoration but rather older age could predict that of work maintenance. In the meta-analysis (3), the predictors were identified mainly in observational cohort studies without interventions, such as a rework program. Therefore, the difference in the patients’ background (i.e., with or without a rework program) might have brought about such a discrepancy. On the other hand, Hoaki and Terao (7) showed that neither age nor gender could predict the probability of work restoration, but the male gender could predict that of work maintenance in patients graduating from a rework program. The reason for this discrepancy regarding the involvement of gender in work maintenance is uncertain; however, it seems likely that the adjustment for cognitive factors, such as the TMT-B score, and mental factors, such as the HAM-D score, might have corrected the apparent deviation induced by age and gender, thereby revealing the true involvement, if any, of age and gender in our study.

Through the ROC curves of the TMT-B scores in relation to work restoration, we determined cut-off points of 66.8 and 76.5 s for the whole sample and restoration group, respectively. Accordingly, we propose a cut-off point of 70 s in the TMT-B completion time for predicting work restoration as “Yamashita’s criterion.” It seems useful to use the criterion for predicting the probability of work restoration during the course of a rework program.

Hori et al. (16) showed that lower SASS scores, lower 3-back correct response rate (N-back test), and higher benzodiazepine dosage were associated with further episodes of sick leave. Moreover, Atake et al. (17) revealed that the cut-off point was 30/31 for the SASS score, 50% for the correct response rate of the three-back task, and a 7.5-mg dose of diazepam equivalent for the use of a benzodiazepine, although their patients did not receive a rework program. However, it seems worthy of further investigating which cognitive test may be useful for predicting the outcome of restoration.

The limitations of this study include the relatively small sample sizes and lack of a control group that did not participate in the rework program. As another limitation, 30% were bank staffs and there were no patients who were lesser qualified and doing manual jobs in our study, which may be deviated from many re-work institutions. Further, as mentioned above, the duration and frequency of participation in our program depend on the status of individual participants, which is comprehensively assessed by staff members; however, the duration in another program is strictly 3 months for all patients (18). Such differences should be considered when evaluating the effects of rework programs and predictors identified in future studies. Finally, further studies are required to validate the Yamashita’s criterion in other groups of patients. Hopefully, international comparisons will be performed to generalize our findings in the near future.

In conclusion, the present findings suggest that unemployed patients rather than patients absent from work may face difficulties in getting a new job and maintaining work, that TMT-B scores can predict the probability of work restoration and maintenance, and that the frequency of rework program participation can predict work restoration but not maintenance.

## Data availability statement

The raw data supporting the conclusions of this article will be made available by the authors, without undue reservation.

## Ethics statement

The studies involving human participants were reviewed and approved by the Oita University Ethics Committee. The patients/participants provided their written informed consent to participate in this study.

## Author contributions

HY and TT planned this study, mainly analyzed the data, and wrote the manuscript. HY and AS were actual working members and collected the data. AS checked the manuscript. All authors read and approved the final manuscript.
